# Revisiting edible insects as sources of therapeutics and drug delivery systems for cancer therapy

**DOI:** 10.3389/fphar.2024.1345281

**Published:** 2024-02-02

**Authors:** Barnali Sinha, Yashmin Choudhury

**Affiliations:** Department of Biotechnology, Assam University, Silchar, India

**Keywords:** edible insects, insect extract, ethnomedicine, anticancer effects, immunomodulants, nanoparticles

## Abstract

Cancer has been medicine’s most formidable foe for long, and the rising incidence of the disease globally has made effective cancer therapy a significant challenge. Drug discovery is targeted at identifying efficacious compounds with minimal side effects and developments in nanotechnology and immunotherapy have shown promise in the fight against this complicated illness. Since ancient times, insects and insect-derived products have played a significant role in traditional medicine across several communities worldwide. The aim of this study was to inspect the traditional use of edible insects in various cultures and to explore their modern use in cancer therapy. Edible insects are sources of nutrients and a variety of beneficial substances with anticancer and immunomodulatory potential. Recently, insect derived bioactive-components have also been used as nanoparticles either in combination with chemotherapeutics or as a nano-cargo for the enhanced delivery of chemotherapeutic drugs due to their high biocompatibility, low bio-toxicity, and their antioxidant and anticancer effects. The crude extracts of different edible insects and their active components such as sericin, cecropin, solenopsin, melittin, antimicrobial peptides and fibroin produce anti-cancer and immunomodulatory effects by various mechanisms which have been discussed in this review.

## 1 Introduction

The development of cancer involves the acquisition of hallmark capabilities and enabling characteristics which are essential for the formation of malignant tumors (Hanahan, 2022). The neoplastic micro-environment which is crucial for the development, expansion, and metastasis of malignancies also contains potential therapeutic targets (Xiao and Yu, 2021). Finding efficient treatment methods are crucial since it is predicted that in the next 20 years, the number of newly diagnosed cancers will rise by approximately fifty percent, globally (Mao et al., 2022).

The common approaches for tackling human cancers are chemotherapy, surgery, radiotherapy and immunotherapy, among which chemotherapy and radiotherapy remain the most widely used. However, these therapies have a wide variety of undesirable side effects, including systemic toxicity, psychiatric issues, as well as the development of highly drug-resistant tumor cells. While chemotherapeutics continue to be widely used, they are not selective towards cancer cells, because of which a significant percentage of healthy cells are also annihilated during the treatment (Liu et al., 2015). Indeed, the most difficult aspect of treating cancer is to eliminate a tumor while sparing healthy cells. However, there have been significant advancements in overcoming the constraints of traditional cancer therapy in recent years (Debela et al., 2021) with folk medicine eliciting much interest in the development of modern medicines which have significantly influenced cancer therapeutics (Yuan et al., 2016). Nature-derived nutraceuticals are garnering great interest due to their structural flexibility which enables them to interfere with crucial signaling components of neoplastic cells, effectively hindering cancer hallmarks (Newman and Cragg, 2020). There have also been concerted efforts towards the discovery of novel peptides from natural sources in order to combat the side effects of chemotherapy and radiotherapy (Dutta et al., 2019). Peptides isolated from natural sources have several benefits over small molecules and biological agents, such as reduced production costs, better tumor tissue penetration, lower immunogenicity and toxicity, binding specificity to the targets, and more adaptable sequences (Abbas et al., 2019; Zhang Y. et al., 2023). Another area of interest has been the discovery of specific nature-derived nano-delivery systems to combat drug resistance as well as adverse side effects (Patra et al., 2018).

Arthropoda is the largest phylum in the animal kingdom, comprising approximately 80% of all living organisms. Class insecta under this phylum possesses vast diversity. Insects serve as sources of medicinal products, therapeutics, and recycling of biological material. Entomophagy, the consumption of edible insects due to their high nutritional value, taste, or associated religious beliefs, has been practiced in many regions of the world since ancient times, particularly in nations like China, Thailand, India, Africa, Latin America, and Mexico (Raheem et al., 2019). Insect extracts have been used as components of folk medicines for various diseases like flu, colds, infections, flatulence, and spasms (Ratcliffe et al., 2011). Insects are also rich sources of proteins, vitamins, chitins, essential amino acids, polyunsaturated fatty acids, and minerals (Oonincx et al., 2010; Boland et al., 2013).

Developing innovative and environment-friendly approaches for new food and drug sources through the effective use of natural resources is amassing worldwide interest (Newman and Cragg, 2020). Insects generate food items and by-products that have a variety of nutritional and practical values, as well as disease-ameliorating properties (Quah et al., 2023). Thus, in recent years, interest in consumption of insects as food has increased due to their established health benefits. There is also increasing interest in the use of insect derived products such as silk, and the silk proteins fibroin and sericin as biomaterial for the formulation of novel nanoparticles and drug delivery systems, owing to their biocompatibility, biodegradability, non-toxicity and low immunogenicity. The silk-based nanoparticles are loaded with chemotherapeutics, natural drugs like curcumin, peptides and proteins, and have also been used for the delivery of nucleic-acid based therapeutics like small interfering RNA (siRNA), micro RNA (miRNA) and antisense oligodeoxynucelotides (ASO) (Florczak et al., 2020).

This review focuses on the role of natural products derived from insects as anticancer therapeutics, and also summarizes their more recent role in the formulation of nanoparticles and nano-cargoes for effective cancer therapy.

## 2 Uses of insects and insect-derived compounds in traditional medicine

Over 2,100 kinds of insects have been categorized as edible, and it is believed that over 2.5 billion people, primarily in Asia, Africa and Latin America ingest insects in various ways (Zhang E. et al., 2023). The indigenous people of several nations such as South America, India, Mexico, Korea, China and Nigeria have a long history of treating ailments using insects (Devi et al., 2023). Lepidoptera are the most commonly used therapeutic insects in Japan, Hemiptera and Orthoptera in India, and Coleoptera, Hymenoptera, Orthoptera, and Homoptera in Brazil (Costa-Neto, 2005). Traditional Chinese Medicine (TCM) has utilized silkworms and flies for healing infected wounds (Sherman et al., 2000; Costa-Neto, 2005) for at least three thousand years (Zimian et al., 1997). Approximately 77 species of insects are also reported to be used in TCM for anti-tumor effects. These include cantharis *Mylabris spp*., caterpillar, bees, wasps, silkworm *Bombyx mori*, house fly *Musca domestica*, ants and grubs (Feng et al., 2009). In certain regions of Brazil, adding ground ants to coffee or juice mixed with sugar is used to alleviate eye ailments (Costa-Neto, 2002). Apitherapy involves employing bee products for therapeutic purposes, which is the most common traditional therapy used by indigenous people for the treatment of cold, cough, stomach pain and fever. Numerous antibacterial peptides and proteins, including cecropins, defensins and lysozymes, are produced by butterflies (Siddiqui et al., 2023). In sub-Saharan Africa, numerous insects are consumed as food supplements because of their high protein and essential amino acid content. They are also used to treat flu, asthma, bronchitis, whooping cough, tonsillitis, sinusitis, and hoarseness (Jideani and Netshiheni, 2017). Insect derived products are frequently used in the Indian traditional medicine system of Ayurveda for treatment of various diseases including anemia, asthma, rheumatism, malaria and ulcer (Wilsanand et al., 2007).

## 3 Anticancer effects of insect derived nutraceuticals in ethnomedicine

### 3.1 Insect extracts

Insects of the Helophoridae family are commonly employed in traditional medicine in Central Asia and Africa. *In vitro* studies on the prostate cancer cell line PC-3 indicated the antioxidant and anticancer activities of protein extracts of *Helophorus aquaticus and Helophorus syriacus*. The protein extracts of both insects showed high efficacy in cell inhibition and produced significant apoptosis at a dose 1,000 μg/mL (Elhazar et al., 2023). Several studies have reported the anti-neoplastic activity of extracts of *Periplaneta americana* which belongs to order Dictyoptera and family Blattidae (Zhao et al., 2017). The isolated components of *P. americana, viz.* “Xiaozheng Yigan Tablet,” “Kangfuxin Liquid,” “Ganlong Capsule,” and “Xinmailong Injection” have been used in TCM. Xiaozheng Yigan is reported to show potent anti-tumor and anti-bacterial effects (Zhao et al., 2017). The impact of *P. americana* on immunological control and anti-tumor activity has also drawn considerable interest. Several *in vitro* studies on human cancer cell lines have reported the anti-tumorigenic activities of the extract as well as isolated components of *P. americana*. It controlled proliferation, downregulated the overexpression of C-erbB-2 and upregulated p53 expression in tumor cells (Zhang and Zhu, 2015). It induced apoptosis by upregulating death ligand Fas and receptor FasR in a lung carcinoma cell line (3LL) while downregulating Bcl2 expression (Jiang et al., 2006). In another study, *P. Americana* extract induced apoptosis through the mitochondrial dependent pathway by reducing mitochondrial membrane potential (Zhao et al., 2017). Cyclohexane extract of *P. americana* L. lysate showed anti-cancer effects on MCF-7 breast cancer cell line whereas no cytotoxic activity was recorded against normal MRC-5 human lung-cells. The lysate could inhibit proliferation and reduce viability of tumor cells in a dose-dependent manner, with an IC_50_ value of 30.2 ± 1.62 μg/mL, whereas its cytotoxicity for normal cells was low, with a CC_50_ value of 118 ± 3.4 μg/mL, which is much higher than its IC_50_ value (Amin et al., 2022), thus indicating a high therapeutic index.

Silkworms are well-known lepidopteron entomophagous insects with high nutritional value and disease ameliorating properties. There are several varieties of silkworms but *Bombyx mori, Antheraea pernyi, Antheraea yamamai, Samia ricini, Antheraea mylitta, Antheraea royle* and *Philosamia cynthia* are the most commonly utilized commercial varieties in the silk industry and for research (Sheikh et al., 2018; Shukurova et al., 2021). Several *in vivo* and *in vitro* studies have reported their antioxidant, anti-inflammatory and anti-cancer activities. The protein extract and oil of silkworm pupae act as anti-neoplastic agents by inducing apoptosis and cell cycle arrest*. In vitro* studies showed that protein hydrolysates extracted from silkworm pupae upregulated the pro-apoptotic proteins Bax and Bak, while downregulating Bcl2 expression. Pupae extract has also been reported to disrupt the mitochondrial membrane potential and induce apoptotic flux in cancer cells (Zhou et al., 2022).

Silkworm protein extracts exhibit anti-tumorigenic properties both *in vitro* as well as *in vivo*. *Bombyx mori* protein hydrolysates exerted anti-proliferative, cell cycle arresting and pro-apoptotic effects on the human gastric cancer cell line, SGC-7901 (Li et al., 2018). It also exerted anti-tumorigenic effects on MGC-803 gastric cancer cell line by impacting metabolic energy supply and inducing cellular organelle rupture (Li et al., 2022). Pupae proteins extracted from *Bombyx mori* and *Samia. ricini* acted as anticancer agents in the breast cancer cell line, MCF-7, by downregulating the inflammatory cytokines IL-6, *IL*-1β and TNF-α (Chukiatsiri et al., 2020), and induced apoptosis in lung, cervical and prostate cancer cell lines (Cho et al., 2019). Selenium-rich amino acid extract of pupae of *Ziyang Sp.* inhibited cell viability and induced apoptosis in human hepatoma cell line through reactive oxygen species (ROS) production (Hu et al., 2005). Fermented silkworm extract induced caspase dependent and independent apoptosis, cell cycle arrest and DNA fragmentation in the hepatocellular carcinoma cell line, HepG2 (Cho et al., 2019). Silkworm (*Bombyx mori*) pupa protein (SPP) produced *in vivo* antitumor activity in colon cancer nude mice by reducing inflammation, inhibiting proliferation and metastasis, and inducing apoptosis (Ji et al., 2022; Zhou et al., 2023).

One of the most popular edible insect species worldwide is mealworm larva (MWL) (*Tenebrio molitor*) belonging to order Coleoptera and family Tenebrionidae. MWL extract showed cytotoxic activity against prostate cancer (PC-3 and 22Rv1), cervical carcinoma (HeLa), hepato-carcinoma (PLC/PRF5, HepG2, Hep3B, and SK-HEP-1), colon (HCT116), lung (NCI-H460), breast cancer (MDA-MB231), and ovarian cancer (SKOV3) cell lines by reducing proliferation and inducing apoptosis, necrosis and autophagy (Lee et al., 2015). Aqueous extract of MWL and *Anoplophora chinensis* showed anti-inflammatory activity against the colorectal adenocarcinoma cell line, Caco-2, and human hepatocellular carcinoma cell line, HepG2, and induced apoptosis by upregulating death receptor and caspase 3 expression (Ding et al., 2021). *In vivo* studies indicate that the larvae and pupae extract of MWL showed anti-proliferative activity against early hepatocellular carcinoma (HCC), while the adult insect extract did not produce any significant changes (Zepeda-Bastida et al., 2021). The anti-proliferative efficacy of MWL oil is attributed to the presence of high concentrations of oleic acid, palmitic acid, and omega-3 fatty acids (Wu et al., 2020).

Insects belonging to order Orthoptera are the fourth most popular edible insects worldwide. The rice field grasshopper, *Oxyachinensis sinuosa* (OCS), is an entomophagous insect and *in vitro* and *in vivo* studies indicate that OCS protein extract exhibited anticancer immunomodulatory activity. It enhanced the maturation of dendritic cells and expression of surface markers such as CD80, CD86, MHC-I, MHC-II on dendritic cell. It also enhanced the differentiation of Th1 cells and CD8^+^ T cells by modulating the NF-Κβ and MAPK pathways (Kim et al., 2020). *In vivo* studies on Kunming mice reported that grub extract from *Holotrichia diomphalia* larvae administered by oral gavage at a dose of 3.9 g/kg and 7.8 g/kg for 10 days inhibited the S_180_ tumor growth, while its LD_50_ dose at 5 days was 48.73 g/kg (Song et al., 2014).

The findings of other *in vitro* studies on the anticancer efficacy of edible insect extracts are given in Table 1.

**TABLE 1:** Anticancer activity of extracts of edible insects.

Insect	Order and family	Experimental model	Result	References
*Gryllus bimaculatus*	Order-Orthoptera	H460, A549 human non-small lung cancer cell	Induced apoptosis through caspase and Bcl2 mediated pathway	Lim and Byun (2021)
	Family- Gryllidae			
*Zophobas morio*	Order-Coleoptera	MCF-7	Inhibited cell proliferation of MCF-7 breast cancer cells while exerting no cytotoxic effects against HUVEC normal cell	Darbemamieh and Soltani (2021)
	Family-Tenebrionidae			
*Vespa orientalis*	Order-Hymenoptera	MCF-7	Cytotoxic effects against MCF-7 cells whereas no cytotoxicity against normal cells. Shows antioxidant activity and induced apoptosis by elevating Bax, Bak, p53 expression and reducing Bcl2 expression. Also inhibits migration of MCF-7 cells	Zedan et al. (2021)
	Family-Vespidae			
*Ulomoides* *dermestoides*	Order-Coleoptera	Human lung cancer A549 cell line	Induced DNA damage to A549 cells and reduced cell viability; benzoquinones isolated from the extract is mainly responsible for genotoxicity and cytotoxicity	Crespo et al. (2011)
	Family-Tenebrionidae			
Ulomoides dermestoides	Order-Coleoptera	HaCaT cells	Phenolic extract shows cytotoxic and genotoxic effects against HaCaT cells	Mendoza-Meza and España-Puccini (2016)
	Family-Tenebrionidae			
*Holotrichia diomphalia* larvae	Order-Coleoptera	*in vitro*- HeLa cells	Grub extract of the insect larvae induced apoptosis reduced tumor growth	Song et al. (2014)
	Family-Scarabaeidae	*in vivo*- Kunming mice		

### 3.2 Insect derived bioactive-compounds and their anticancer activities

#### 3.2.1 Cecropin

Cecropin is an anti microbial peptide (AMP) found in the hemolymph of insects, which provides innate immunity to the insects. It was originally isolated from the hemolymph of *Hyalophora cecropia* pupae. The peptide is 34–37 amino acids long (sequence: KWKLFKKIEKVGQNIRDGIIKAGPAVAVVGQATQIAK). It lacks cysteine residues and is folded in an alpha helical structure (Hoskin and Ramamoorthy, 2008; Lee et al., 2013; Yang M. et al., 2023). The overall charge of this cationic peptide is +7 at pH 7, and it contains 47% hydrophobic amino acid residues (Yang M. et al., 2023). The amino terminal of the peptide contains both polar and non-polar amino acids which gives it an amphipathic nature whereas the carboxy terminal has hydrophobic amino acids. Cecropin is also derived from other edible insects, namely, *Bombyx mori, Musca domestica, Acalolepta luxuriosa, Helicoverpa armigera, Papilio xuthus* and *Drosophila melanogestar* (Brady et al., 2019; Ziaja et al., 2020).

The cecropin protein family comprises proteins mainly present in holometabolous insects which differ slightly in amino acid sequence, resulting in different cecropins types *viz.,* A, B, C, D and P1 (Ziaja et al., 2020). AMPs belonging to the cecropin superfamily have also been identified from other animal classes, such as, cecropin P from pig and cecropin-like styelin, isolated from tunicates (Brady et al., 2019).

While the anti-microbial properties of cecropin and its analogues are well established, its anti-neoplastic properties are less documented. Cecropin interacts with the negatively charged lipids in the cell membrane of bacteria through its N-terminal helix and creates pores in the membrane through its hydrophobic C-terminal helix, disturbing membrane permeability. The wide spectrum anti-bacterial activity of cecropin is attributed to this interaction with membrane lipid bilayers (Vakili and Jahanian, 2023). The membranes of cancer cells differ in lipid composition from normal cells and have an elevated negative charge due to exposure of phosphatidylserine on the outer leaflets of the membranes. Thus, cationic AMPs like cecropin have a high affinity for interacting with and disrupting the cell membranes of cancer cells by inducing pore formation, while sparing healthy cells (Ziaja et al., 2020) thereby holding great promise as anti-cancer agents. Cationic AMPs have also been reported to disrupt the mitochondrial membranes of cancer cells, to induce apoptosis by downregulating anti-apoptotic genes and upregulating apoptotic genes, and to exert immunomodulatory effects against cancer cells and tumor microenvironment. They also inhibit tumor growth by modulating several signaling pathways involved in cell survival and proliferation (Lei et al., 2019; Jafari et al., 2022). Indeed, Cecropin D, from *Bombyx mori* exhibits pro-apoptotic features and targets esophageal cancer by destabilizing mitochondrial membranes (Ramos-Martín et al., 2022). The N- terminal amphipathic sequence is crucial for the interaction with the anionic lipid components of the neoplastic cell membrane, and the cecropin B3 analog, which does not have the N- terminal fails to induce pore formation (Ye et al., 2004).

Different types of cecropin isolated from *H. cecropia* (cecropin A, B), *B. mori* (cecropin XJ) and *M. domestica* (cecropin Mdc) show potent anti-neoplastic activity in several *in vitro* human and rodent cancer cell lines (Brady et al., 2019). Cecropins in their conjugated forms have also demonstrated anti-tumor action. An amalgamation peptide called CA-ME, which includes 1–12 residues of another AMP, melittin, and 1–8 residues of cecropin A is cytotoxic to small cell lung cancer (SCLC) cell lines. Likewise, another hybrid peptide, CA-MA has been demonstrated to have anticancer effects on SCLC cell lines which contains sequences from cecropin A (1-8 residues) and magainin 2 (1–12 residues) (Shin et al., 1998). Cecropin A shows anti-neoplastic efficacy against human pro myelotic cell line HL-60 by reducing cell viability and inducing cell cycle arrest and cell death in a caspase independent manner. Cecropin A also elevates ROS production in cancer cells and induces DNA fragmentation in a dose dependent manner, ultimately inducing caspase independent cell death (Cerón et al., 2010). An alpha helical cyclic cationic peptide designed from cecropin B which has the same hydrophobicity as cecropin B, was reported as a potent anti-cancer agent against Dalton’s lymphoma ascites (DLA) and Ehrlich’s ascites carcinoma (EAC) cell lines (Sharma et al., 2019). The IC_50_ value of cecropin A and cecropin B against all bladder cancer cell lines ranged from 73.29 μg/mL to 220.05 μg/mL (Suttmann et al., 2008).

Cecropin A in combination with chemotherapeutic drug 5-fluorouracil or cytarabine produced more effective anticancer activity in CCRF-SB lymphoblastic leukemia cells than 5-fluorouracil alone (Hui et al., 2002). In another *in vitro* study, cecropin B coupled with modified leutinizing hormone releasing hormone (LHRH) showed potential anti-neoplastic activity against drug resistant ovarian cancer (SK-OV-3, ES-2, NIH: OVCAR3) and human endometrial adenocarcinoma-1 (HEC-1A) cell lines. However, the combination did not produce any cytotoxic activity against normal cells (Li et al., 2016). Cecropin in combination with other anti-cancer agents like curcumin also resulted in enhanced anticancer effects. Cecropin A levels were elevated in curcumin treated *Musca domestica* hemolymph and also showed higher cytotoxicity, anti-proliferative activity and induced G2/M cell cycle arrest in MCF-7 cells, but was not cytotoxic to normal vero cells (Mahmoud et al., 2022). The M1-8 peptide (G-W-L-K–K-I-G-K) derived from the N-terminal region of cecropin isolated from the hemolymph of *M. domestica* larvae induced lysosomal leakage and lysosome mediated apoptosis in human hepatocellular carcinoma cell line, HepG2, and HepG2 xenograft mouse model (Zeng et al., 2023).

While the C-terminal helix of cationic cecropin is hydrophobic, an anionic cecropin isolated from the larvae of *Choristoneura fumiferana* belonging to the Lepidoptera family is characterized by the presence of L-aspartic acids in the C-terminal region, which make the C-terminus helix amphipathic in nature,. The BH3 like motif (G-[KQR]-[HKQNR]-[IV]-[KQR]) which inhibits Bcl2 to induce apoptosis may be found in both anionic and cationic cecropin (Maaroufi et al., 2021).

Table 2 enlists the anticancer activity of cecropin on different experimental models.

**TABLE 2:** Anticancer efficacy of cecropin on different experimental models.

Compound	Experimental model	Dose	Result	Reference(s)
Cecropin from *Musca domestica*	Human hepatocellular carcinoma cell line, BEL-7402	100 µM	Induced extrinsic apoptotic pathway by upregulating Fas, Fas-L, caspase-8, and caspase-3and thus shows anti-tumor effects	Jin et al. (2010)
Cecropin from *Musca domestica*	Human hepatocellular carcinoma cell line, BEL-7402	50 µM; 100 µM	Anti-tumor effects on BEL-7402 cells by cytotoxic effects	Jin et al. (2014)
Cecropin from *B. mori*	Human esophageal cells, Eca109 and TE13	100 μg/mL	Induced mitochondrial dependent apoptosis by releasing cytC and upregulate caspase3	Xu et al. (2020)
Cecropin from *Musca domestica*	Human hepatocellular carcinoma cell line, BEL-7402	50 µL of crp 24 mg/kg/day mice	Induced apoptosis and produced anti-tumor effects	Jin et al. (2013)
Cecropin A and B	Human bladder cancer cell line RT4; 647V; J82; 486P	IC_50_ value of cecropin A and B against all tested bladder cancer cell lines ranged from 73.29 μg/mL to 220.05 μg/mL	Disrupted the cell membrane and induced cytolysis of the tumor cells; anti-proliferative effects	Suttmann et al. (2008)
Cecropin A	Promyelotic cell line HL-60	30 µM	Inhibited cell viability and induced apoptosis in mitochondrial caspase independent manner; it also triggered ROS generation and exposed phosphatidylserine in the outer membrane	Cerón et al. (2010)
Synthesized cecropin A and its analog	Human myelogenous leukemia (K562), human monoblastic leukemia (U937), and human acute monocytic leukemia (THP-1)	20, 40, 80, 160, 320, 640 µM	Produced anticancer activity by changing cell membrane permeability and cytotoxicity	Sang et al. (2017)
cecropinXJ from *B. mori*	Human gastric carcinoma BGC823 xenograft tumor model	20, 50, 80, 100 μg/mL	Elevated Bax level, downregulated Bcl2 and induced apoptosis in mitochondrial caspase dependent manner; prevented tumor angiogenesis	Wu et al. (2015b)
CecropinXJ from *B. mori*	Human esophageal cell line Eca109	10 µM	Induced cytotoxicity in Eca109 cells through cytoskeleton breakdown and altered expression of cytoskeletal proteins	Xia et al. (2014a)
CecropinXJ from *B. mori*	Hepatocellular carcinoma cells, Huh-7	1–50 µM	CecropinXJ induced apoptosis in Huh-7 cells through caspase 3 and poly (ADP ribose) polymerase. Additionally, cecropinXJ elevated the expression of Bcl2-associated X protein and the Bcl2-associated death promoter while downregulating the expression of B-cell lymphoma 2 (Bcl2) protein	Xia et al. (2016)

#### 3.2.2 Sericin

Sericin is produced by silkworm during the metamorphic stage while transforming from larvae to pupae (Kunz et al., 2016). It is synthesized in the silk gland of the silkworms and acts as an anchoring molecule for the fibroin to form the silk fibers (Dhawan and Gopinathan, 2003). Sericin protein is discarded in the silk industries during the degumming process of cocoons (Gupta et al., 2013). It is a globular glycoprotein consisting of 18 amino acids folded into β-sheet and can be transformed into a randomly coiled structure under specific physiological conditions (Takasu et al., 2007; Kunz et al., 2016). It is highly hydrophilic and predominantly contains serine (40%), aspartic acid, threonine, tyrosine and glycine in high amounts. Alternative splicing of three genes namely *ser1*, *ser2*, *ser3,* results in a high level of molecular heterogeneity of the sericin protein among insects of the Lepidopterian family (Michaille et al., 1989; Kunz et al., 2016). The physiochemical properties of sericin depends on the extraction procedure and the insect source (Seo et al., 2023). It acts as an antioxidant and can neutralize ROS by donating proton/hydrogen ion (Mumtaz et al., 2023), and can counteract oxidative damage induced by hydrogen peroxide (Dash et al., 2008). Several studies have reported its disease ameliorating effects due to its antioxidant and anti-inflammatory properties, making it of potential use in the food and cosmetic industries (Kunz et al., 2016; Seo et al., 2023).

Sericin can interfere with several signaling pathways associated with the hallmarks of cancer and acts as a potent anti-neoplastic agent by inducing apoptosis and cell cycle arrest (Kunz et al., 2016).

*In vivo* studies on a murine model of colon cancer induced by 1,2-dimethylhydrazine reported a 62% reduction of colonic adenoma upon inclusion of sericin in the diet for 115 days through reduction of oxidative stress, suppression of cell proliferation and oncogene inhibition (Zhaorigetu et al., 2001). A diet containing 3% sericin when administered for 5 weeks also exerted anti-tumorigenic effects in colon carcinogenesis induced by 1,2-dimethylhydrazine (Sasaki, 2000). Undigested sericin in the colon reduced colon mucosal lipid peroxidation by 34% and intestinal aberrant crypt foci by 36% (Zhaorigetu et al., 2007). Sericin also exerted protective effects on mouse skin tumorigenesis induced by 12-O-tetradecanoylphorbol (TPA) and 7,12-dimethylbenz (α)-anthracene (DMBA). Sericin protein delayed tumor appearance, decreased inflammatory cytokines and also c-myc, c-fos oncogene production in this model of mouse skin tumorigenesis (Zhaorigetu et al., 2003).

*In vitro* studies on colon cancer cell line SW480 reported that small sized sericin (61–132 kDa) effectively reduced SW480 cell viability and induced apoptosis through caspase-3 activation and suppression of expression of Bcl2. However, it had no apoptotic effects on the normal colon cell line, FHC (Kaewkorn et al., 2012). In an *in vitro* study, sericin extracted from the non-mulberry silkworm, *A. proylei* J., showed apoptotic effects on the human lung cancer cell line, A549; the cervical cancer cell line, HeLa; and the prostate cancer cell line, PC-3. Sericin induced apoptosis in PC-3 and HeLa cell lines by activating p38, and through the phosphorylated ERK pathway in the A549 cell line, thus inducing apoptosis in different cell lines by different mechanisms. Sericin extracted from *A. proylei* J. showed antitumorigenic activity against A549 and HeLa cells at IC_50_ values of 3.8 μg/mL and 3.9 μg/mL, respectively (Devi et al., 2023).

Sericin induced cell autophagy in the gastric cancer cell line, MKN45, and in nude mice xenografted with MKN45 cells. It elevated the expression of autophagy markers beclin and LC3-2, and lowered the expression of p62 in these systems (Guo W.-H. et al., 2018). Sericin from *Bombyx mori*, *Antheraea assamensis*, and *Philosamia ricini* showed anti-cancer effects against A431, SAS, and MCF-7 cell lines. It induced apoptosis by elevating Bax expression while concomitantly downregulating the expression of Bcl-2 (Kumar et al., 2019). Sericin also showed potent anti-neoplastic activity against triple negative breast cancer cell line MDA-MB-468, by causing G0/G1 cell cycle arrest and inducing apoptosis by suppressing PI3K/AKT signaling (Niu et al., 2021).

#### 3.2.3 Solenopsin

Solenopsin is an alkaloid found in the venom of red ants *Solenopsis invicta* and *Solenopsis germinate*. It has a piperidine ring in its structural makeup, with a methyl group substituted at position 2, and a long hydrophobic chain at position 6 (Park et al., 2008; Kachel et al., 2018).

Solenopsin is reported to exert anti-angiogenic effects by inhibiting the PI3K signaling pathway and also potentially inhibiting neuronal nitric oxide synthase (nNOS). The solenopsin analog, compound B (*MU-06-SC-608-7*), inhibits Akt activation by downregulating its phosphorylation at Thr308 in ras-transformed rat liver epithelial cells WBras1, and human lung cancer cells H2009, while suppressing downstream target proteins along the Akt pathway. A reduction in cell viability was noted at doses greater than 5 µM (Uko et al., 2019). The anti-angiogenic activity of solenopsin has been reported *in vivo* in zebra fish (Arbiser et al., 2007). Solenopsin inhibits the phosphorylation of Akt and its downstream transcription factor forkhead box 01a (FOXO1a) (Arbiser et al., 2007; Kang et al., 2019). Table 3 lists the anticancer efficacy of solenopsin on different experimental models. Research on it is still in the early phases, but is encouraging, and solenopsin may 1 day serve as a treatment for various neoplasia. More research is required to confirm its safety and efficacy in human populations.

**TABLE 3:** Anticancer efficacy of solenopsin on different experimental models.

Compound	Experimental model	Dose	Result	References
Solenopsin A	Zebra fish	1 μg/mL, 3 μg/mL, 6 μg/mL	Shows anti angiogenic activity by inhibiting PI3K pathway; it also inhibit the phosphorylation Akt and FOXO1a	Arbiser et al. (2007)
Solenopsin A and its analog *compound B (MU-06-SC-608-7)*	WBras1; H2009 Human carcinoma cell	>5 µM	Anti-tumorogenic effects by inhibiting PI3K and Akt phosphorylation	Uko et al. (2019)

#### 3.2.4 Bee venom

Bee venom or apitoxin is synthesized by bees in their venom gland situated in the abdominal cavity, and is used as a defensive chemical weapon against predators (Kim et al., 2020; Nainu et al., 2021; Ullah et al., 2023). It is a translucent acidic mixture containing several bioactive components including enzymes, proteins, and non-protein parts. Chemicals present in bee venom are reported to possess several disease ameliorating properties. Its therapeutic benefits have been known since ancient times. In ancient medicine, bee venom was used to cure arthritis, rheumatoid arthritis, back ache, and dermatitis (Ullah et al., 2023).

In recent years, several *in vivo* and *in vitro* studies reported that chemical constituents found in bee venom can be used in the treatment of diseases like cancer, arthritis, skin diseases, and diseases associated with the vascular system. Its promising positive effects have been reported against several cancers such as hepatocellular carcinoma, ovarian, prostate, breast, lung and urinary bladder cancer, and melanoma (Wehbe et al., 2019; Salama et al., 2021; Shi et al., 2022). The main constituent of bee venom is melittin which comprises 40%–50% of the total dry weight. It consists of 26 amino acids (^+^H-Gly-Ile-Gly-Ala-Val-Leu-Lys-Val-Leu-Thr-Thr-Gly-Leu-Pro-Ala-Leu-Ile-Ser-Trp-Ile-Lys-Arg-Lys-Arg-Gln-Gln-NH_2_), and is a cationic amphipathic peptide with six positively charged amino acid residues and no negative charges. Most of the positive charges occur at its C-terminal end which is hydrophilic in nature and has lytic activity, whereas the N-terminal region is hydrophobic in nature (Pincus, 2012; Lee and Bae, 2016). It can create ephemeral or stable pores in the cell membrane in a concentration dependent manner (Pino-Angeles and Lazaridis, 2018; Wehbe et al., 2019) and is thus cytolytic (Lee and Bae, 2016). Melittin was shown to have cytotoxic effects on certain cancer cell types. It triggered apoptosis in the leukemia cell line, U937, by blocking Akt signaling. Melittin also inhibited the proliferation of leukemia cells by blocking calmodulin protein. It is reported to inhibit the TLR2, TLR4, CD14, NEMO, and PDGFR signaling pathways while activating the p38, ERK1/2, AKT, and PLC1 pathways, elevating calcium channel activation, activating death receptors (DR4, DR5), and indirectly stimulating the apoptosis-related caspase 3 and caspase 9 enzymes (Wehbe et al., 2019; Abaci, 2022).

*In vitro* studies on HeLa CK and CK2 cells reported that pre-treatment of these cells with melittin enhances the anticancer effects of cisplatin by enabling cisplatin to permeate the cell membrane more easily, thus increasing the cytotoxicity to cancer cells (Gajski et al., 2014). Melittin also retarded the proliferation of colorectal cancer in mice xenograft model by inducing apoptosis due to ER stress and imbalance of calcium homeostasis, while not inducing pathological changes in biomedical and hematological parameters unlike the chemotherapeutic drugs, cisplatin and 5-flurouracil (Luo et al., 2023). Melittin inhibited cell viability and induced apoptosis by elevating Ca^2+^ and Zn^2+^influx and inducing mitochondrial reactive free oxygen species (MitSOX) in the glioblastoma cells. Melittin enhanced the anticancer effects of cisplatin in the glioblastoma cells,DBTRG-05MG, through TRPM2 mediated apoptosis (Ertilav and Nazıroğlu, 2023).

Melittin has been shown to have anti-neoplastic effects on lung cancer cells by lowering the protein expression of vascular endothelial growth factor (VEGF) and hypoxia-inducible factor 1 (HIF-1) (Zhang and Chen, 2017). The demethylation of the PTCH1 (protein patched homologue 1) promoter may be induced by melittin, increasing the expression of PTCH1. Additionally, melittin treatment dramatically decreased the expression of sonic hedgehog (Shh) and human glioma-associated oncogene homolog 1(GLI1). Indeed, melittin reduced cell growth in SMMC-7721 cells by lowering methyl CpG binding protein 2 (MeCP2) through Shh signaling (Wu X. et al., 2015).

The ADAMTS (A Disintegrin And Metalloproteinase with Thrombospondin Motifs) family stimulates or inhibits the tumorigenic capacity of tumor cells through changes in the cancer microenvironment, (Cal and López-Otín, 2015; Kelwick et al., 2015). Long non coding RNA ADAMTS9 antisense RNA 2, ADAMTS9-AS2, plays a critical role in neoplasia by suppressing cancer metastasis (Liu D. et al., 2020). *In vitro* studies on MHCC97-H and HepG2 cell lines revealed that melittin elevated the expression of ADAMTS-AS2 via downregulating DNA methyl transferase protein-1 (DNMT1) protein, which causes demethylation of ADAMTS-AS2 promoter. ADAMTS-AS2 subsequently hindered the Akt signaling pathway to produce anti-cancer effects (Lv et al., 2023).

Graphene nanoparticles used to deliver melittin to the breast cancer tumors grown on chorioallantoic membrane from chicken embryos resulted in more potent anti-neoplastic activity than melittin alone, by elevating the cytotoxic effects of melittin, increasing cytokine secretion and inhibiting tumor progression (Daniluk et al., 2023). A hybrid peptide designed by conjugating TAT (RKKRRQRRR) and a peptide from the N-terminal region of melittin (GLPAL- ISWIKRKRQQ) possesses high potency of penetration into cancer cells. *In silico* studies with this peptide revealed that it has higher binding efficacy with CD147 and CypA proteins and inhibits their interactions. The CypA/CD147 interaction is a crucial route in some cancer types and is also necessary for the COVID-19 virus to infect the host cell. Thus, it is suggested that the designed peptide may prove to be an appealing treatment target for controlling different tumor types as well as COVID-19 infection (Maani et al., 2023). Melittin showed selective cytotoxicity against HER2-enriched breast cancer cell lines (MDA-MB-453 and SKBR3) and Triple Negative Breast Cancer (TNBC) cell lines (SUM 159 and SUM149), while exhibiting minimal cytotoxicity against normal cell lines (HDFa, and MCF 10A and MCF-12A cells) (Duffy et al., 2020). While the *in vitro* anti-cancer effects of melittin are well established, more *in vivo* studies are required to elucidate its systemic toxicity and cytotoxicity against normal cells.

## 4 Possible anticancer mechanisms of bioactive compounds isolated from edible insects

Anti-cancer therapeutics can exert their effects through various mechanisms such as by influencing the genes that regulate the cell cycle, by inducing apoptosis, or by inhibiting proliferation. The *phosphatidylinositol 3-kinase (PI3K)/protein kinase B (Akt)* pathway is an important signaling pathway controlling the proliferation and survival of cells. Malignant neoplasia such as breast, lung, ovarian, and prostate tumors exhibit aberrant activation of this pathway. Increased activity of the pathway is frequently linked to the development of tumors and resistance to cancer treatments. A potential therapeutic agent can thus produce anti-cancer effects by modulating the intermediates of the PI3K-AKT pathway (Yang et al., 2019; Liu R. et al., 2020). Cecropin inhibits Bcl2 through its BH3 like motif and also upregulates pro-apoptotic proteins (Jin et al., 2010; Maaroufi et al., 2021). As forkhead box O proteins (FOXOs) build up in the nucleus, they can bind to different transcriptional cofactors and control the expression of genes involved in the growth of cells, survival, proliferation, cell division, apoptosis and metabolism. The primary route controlling the transcriptional activity of FOXOs is the PI3K/Akt pathway (Burgering and Medema, 2003). Solenopsin is reported to exert anti-cancer effects by inhibiting Akt phosphorylation and activation of its downstream protein, FOXO1 (Arbiser et al., 2007; Uko et al., 2019). Sericin induces apoptosis via caspase dependent pathway, and enhances expression of the pro-apoptotic proteins Bax, Bim, Puma and Noxa by inhibiting the anti-apoptotic protein, Bcl2 (Kaewkorn et al., 2012; Kumar et al., 2019). It also induces p53-dependent apoptosis in cancer cells (Jolly Devi et al., 2023).

**FIGURE 1:**
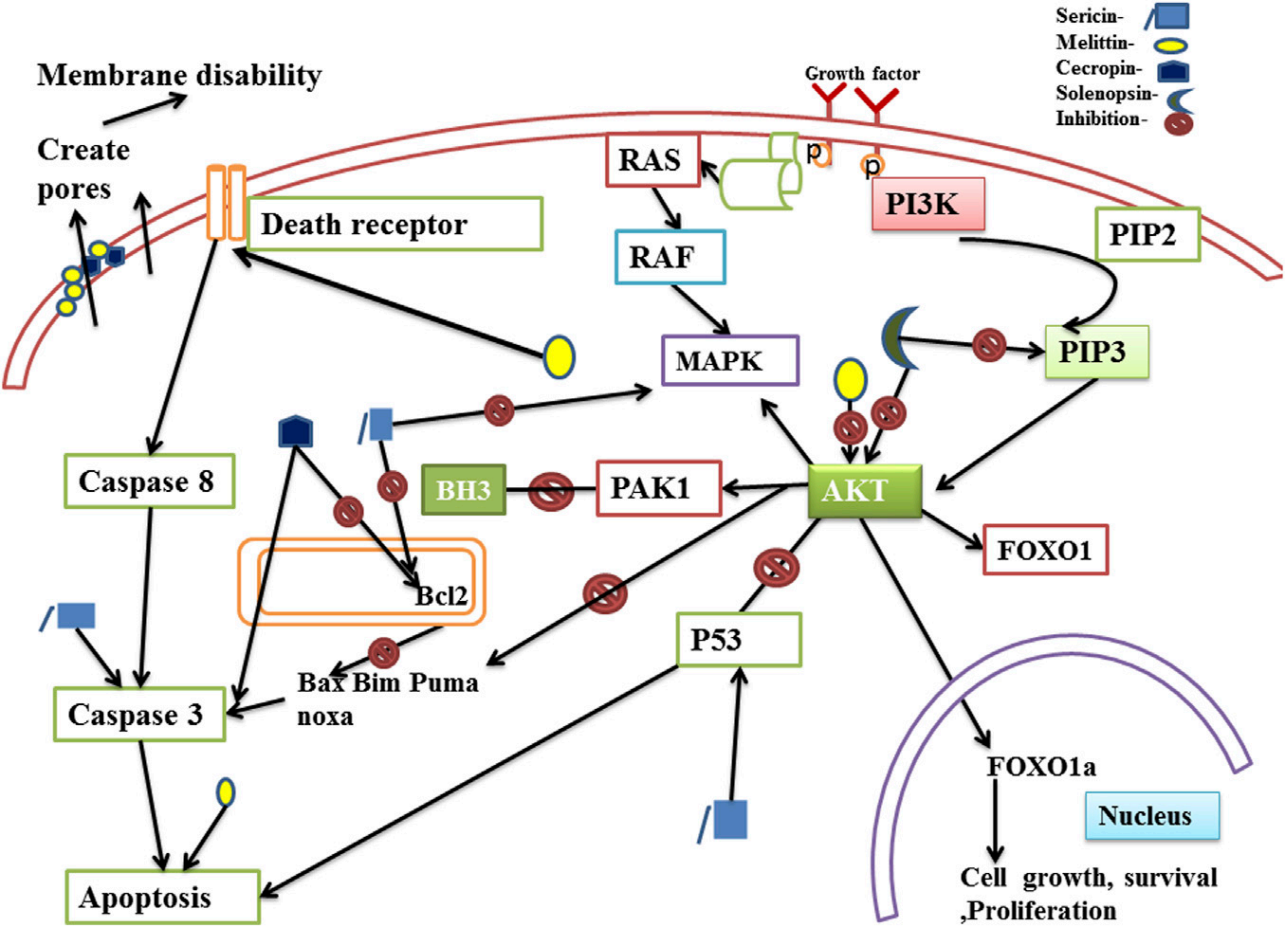
Anticancer mechanism of compounds isolated from insects through the PI3K/Akt pathway. The PI3K/Akt pathway actively promotes carcinogenesis in metastatic tumor cells. Activated phosphoinositide 3-kinase (PI3K) phosphorylates Phosphatidylinositol 4,5-bisphosphate (PIP2) and converts it to Phosphatidylinositol 3,4,5-trisphosphate (PIP3) which further phosphorylates protein kinase B (Akt). Fully activated Akt exerts a variety of downstream effects on signaling molecules. Akt phosphorylates several downstream target genes and proteins which helps to prevent apoptosis, autophagy and promote cell survival, cell growth and proliferation. Insect derived bioactive compounds namely sericin, solenopsin, melittin, cecropin can induce apoptosis and inhibit cell proliferation by modulating the PI3K/Akt pathway to produce anticancer effects.

The anticancer mechanisms of insect-derived active components sericin, cecropin, solenopsin and melittin have been depicted in Figure 1.

**FIGURE 2:**
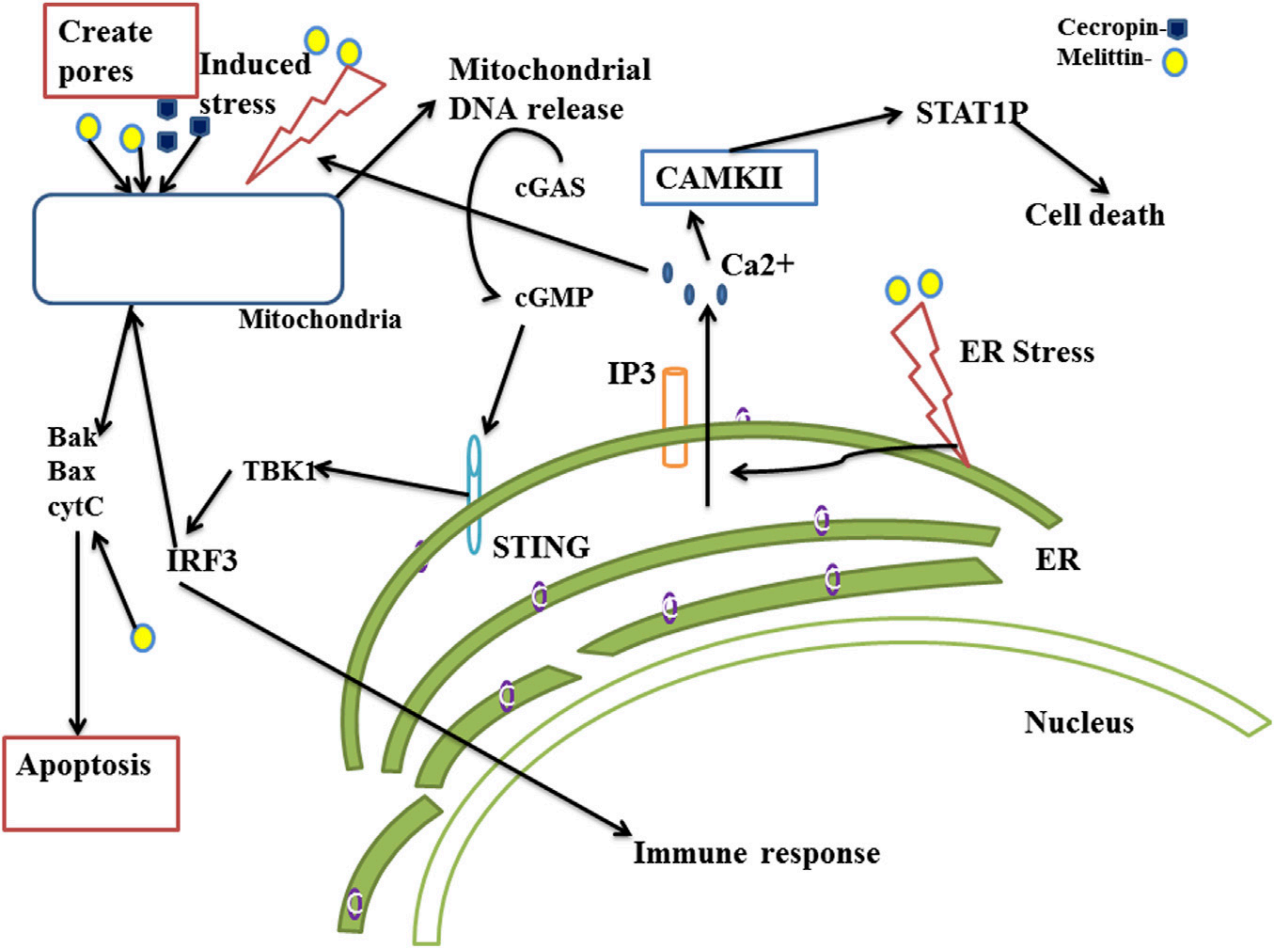
Anticancer mechanism of melittin and cecropin through cyclic GMP–AMP synthase (cGAS) stimulator of interferon gene (STING) (cGAS STING) and endoplasmic reticulum (ER)-mitochondrial stress mediated cell death. The cGAS-STING system, also known as the cyclic GMP-AMP synthase (cGAS)-stimulator of interferon genes (STING) route, works as a cytosolic DNA sensing mechanism that initiates innate immunity and inflammatory responses. The DNA sensor enzyme, CGAS, catalyzes the production of cyclic GMP-AMP (cGAMP), which binds to STING and activates downstream signaling molecules. The cGAS-STING pathway is activated, which increases the production of type I interferons and pro-inflammatory cytokines and strengthens the immune system of the host against viral infections, cancer, and autoimmune illnesses. STING activates its downstream molecules TBK1 and IRF3. Activated IRF3 performs dual functions, triggers the release of apoptotic proteins bax, bak and also moves to the nucleus to induce the release of INF-I and pro-inflammatory cytokines. Melittin and cecropin disrupt the mitochondrial membrane potential dynamics, mediate membrane pore formation and induce mitochondrial dependent apoptosis and cGAS-STING pathway mediated antitumor response. They also induce ER stress and which results in release of Ca^2+^ and induces mitochondrial pore formation. Melittin induces ER stress and results in ER mediated apoptosis.

The cGAS-STING pathway regulates several pathological processes brought on by the immunological response to the ectopic localization of self-DNA, including cytosolic mitochondrial DNA, in addition to protecting cells against a variety of DNA containing pathogens. Besides its well established antimicrobial properties, melittin creates pores in the cell membrane and the mitochondrial membrane, and may lead to exposure of mitochondrial DNA in the cytoplasm of cancer cells (Díaz-Achirica et al., 1994; Wang et al., 2022). It also causes morphological changes in the cell membrane and DNA damage in leukocytes even at non-cytotoxic doses (Guha et al., 2021). The presence of cytosolic DNA and DNA damage are detected by cyclic GMP-AMP synthase (cGAS) which activates the stimulator of interferon genes (STING), leading to cGAS-STING mediated cell death, induction of type-I interferons and cytokine production, ultimately inducing apoptosis and anti-tumor immune activation. The generation of type I interferons by the stimulation of the cGAS-STING pathway has the potential to significantly enhance anti-tumor immunity (Gan et al., 2022). It is reported that MnO2-melittin nanoparticles activate the cGAS STING pathway to produce anticancer activity (Tang et al., 2022). ER stress leads to release of Ca^2+^ ions to the cytosol which trigger mitochondrial membrane permeability and pore formation, resulting in leakage of mitochondrial DNA to the cytosol (Smith, 2021). Melittin induces ER stress and release of Ca^2+^ through IP3, thus inducing ER mediated apoptosis (Luo et al., 2023). The anticancer mechanisms of melittin and cecropin through the cGAS STING pathway have been illustrated in Figure 2.

## 5 Immunomodulatory effects of edible insect extracts and active components on cancer

Inflammation is intimately linked to all phases of the onset and spread of malignancy in the majority of cancers, as well as to the effectiveness of anti-cancer treatments (Bader et al., 2020; Arner and Rathmell, 2023). The immune system is assisted by immunotherapy in identifying and eliminating cancer cells. By combining vaccinations with immunostimulatory cytokines or by inhibiting the mechanisms that cancer cells employ to dampen the response, immunotherapy seeks to increase the immune system’s ability to fight cancer (Markman and Shiao, 2015). Immunomodulation for the treatment of cancer involves two factors: (1) enhancing the immune system’s capacity to combat cancer by turning on anti-cancer immune cells (2) Blocking pro-cancer immune cells or causing them to polarize towards anti-tumor types by focusing on essential signaling pathways which can slow the development of cancer (Locy et al., 2018).

Various studies have reported that insect derived bioactive compounds and insect extracts possess immunomodulatory activity. A coleopteran insect *Mimela sp*. belonging to Scarabaedae family is an entomophagous bug mostly consumed in Korea, Northern Thailand, and the state of Arunachal Pradesh in India. Studies reported that it has antioxidant, immunomodulatory and anti-tumorigenic activities. *Mimela sp*. extract elevated the leucocyte count in cyclophosphamide treated mice. It also increased TNF-α and IL-6 in immunosuppressed mice (Tukshipa and Chakravorty, 2022). *Eupolyphaga sinensis* Walker is a wingless edible cockroach belonging to order Blattaria and the family Polyphagidae. *In vivo* studies reported that *E. sinensis* extract enhances immunity in mice, improves lymphocyte production and also stimulates T-cell mediated delayed type hypersensitivity (Tang et al., 2010). Lectins isolated from the edible insect pupae of *Musca domestica* belonging to family Muscidae show immunomodulatory and anti-tumor effects. Three galactose specific lectins (40kDa, 55kDa and 80 kDa) show potent immunomodulatory activity on murine peritoneal macrophages, enhancing the phagocytic activity of macrophages via NF-kB pathway and increasing the expression of TNF-α, IL-6 and INF-γ (Cao et al., 2012). *In vivo* studies on mice model revealed that freeze dried *Tenebrio molitor* larvae enhance the phagocytic activity of macrophages in mice. It produces immunomodulatory effects by intensifying the non-specific, cellular and antibody mediated immune responses (Tang and Dai, 2016).

*In vivo* studies on mice injected with sarcoma S180 cells revealed that a peptide fraction extracted from *Musca domestica* larvae showed immunomodulatory activity by encouraging the growth of splenocytes, NK and CTL activity, and boosting serum levels of IgG, IgG2a, and IgG2b antibodies that are specific for the antigen in S180 sarcoma cells in mice. Additionally, the peptide fraction elevated the mRNA expression of INF-γ, and promoted Th1 response by elevating the expression of transcription factor T-bet and STAT-4 in sarcoma S180 cell bearing mice (Sun et al., 2014). Bee products are well known for their antioxidant, anti-inflammatory and anti-tumor effects. Recent *in vivo* and *in vitro* studies reported the immunomodulatory effects of bee pupae peptide, BPP-22. It enhanced the production of antibodies IgA, IgE, IgM, IL-2, INF- γ and elevated the phagocytic activity of macrophages in an immunosuppressied mouse model. It is suggested that BPP-22 exerts its immunomodulatory effects by elevating the phosphorylation of ERK and p38 and modulating the MAPK signaling pathway (Chen et al., 2022).

Mellitin has been reported to have a range of immune-modulating effects in several studies. Melittin alone or in conjugation with other anticancer peptides enhanced the secretion of IL-2 and TNF-α, elevated the proliferation of splenocytes and cytotoxicity of NK cells, enhanced Th1 specific INF- γ production, and increased the activity of macrophages. Melittin is also used as a tumor vaccine *in vitro* in conjugation with other components to induce the immune reaction and enhance the activity of dendritic cells to kill melanoma cells. Injectable hybrid vaccine hydrogel was prepared by conjugation of melittin, RADA32 (cell assembling peptide), CpG (immune adjuvant) and tumor lysate. This hybrid vaccine showed anticancer activity *in vivo* by elevating cytotoxic T lymphocytes (CTLs) and activating dendritic cells in draining lymph nodes in melanoma B16-F10 xenograft mice (Yang K. et al., 2023).

**FIGURE 3:**
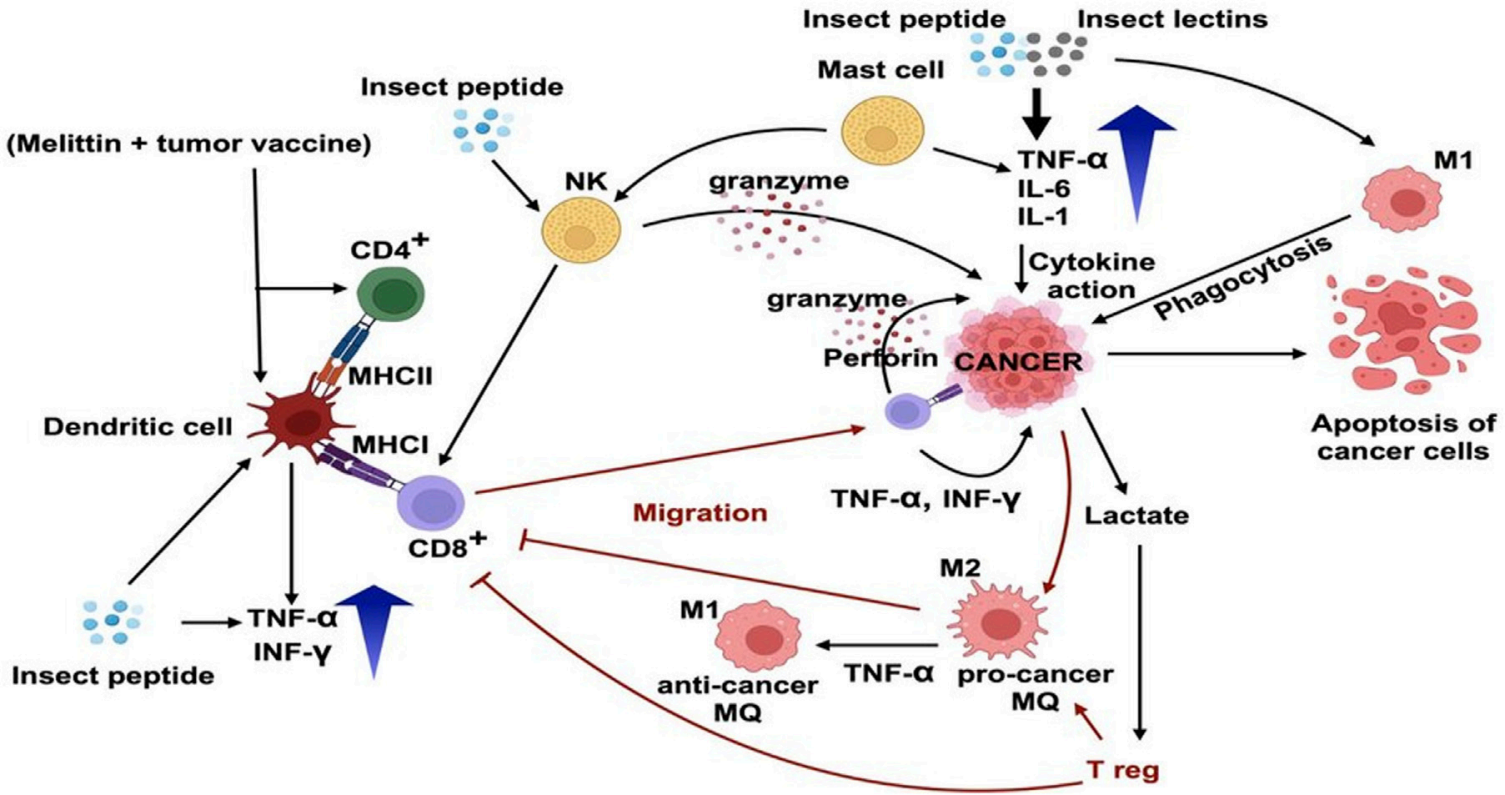
Immunomodulatory effects of insect (*M. domestica; Mimela sp.; E. sinensis; P. vicina*) peptides and *M. domestica* derived lectins, as well as melittin on tumor cells and tumor microenvironment. Melittin in combination with tumor vaccine and insect peptides stimulate the dendritic cells (DCs) and maturation and migration of CD8^+^ T cells to the tumor site. Several insect peptides produce anticancer effects by activating M1 anticancer macrophages which show cytotoxic effects against cancer cells. Insect peptides and lectins isolated from insects elevate TNF-α, IL-6, IL-1 and induce NK cells to release granzymes. The image was created using Biorender.Com.

The immunomodulatory activity of different insect peptides against cancer is depicted in Figure 3. Dendritic cells (DCs) undergo functional morphological modification and activate T helper cells (T CD4^+^) and T cytotoxic cells (T CD8^+^). T helper cells release cytokines and other mediators that control other immune cells. These cytokines activate and polarise monocytes and macrophages as well as control the antibodies produced by B cells. As a result, Th cells are essential to the anti-cancer immune system. On the other hand, the T cytotoxic cells (T CD8^+^) migrate towards the cancer cells and directly destroy them by releasing granzymes and perforins. NK cells also enhance the maturation of T CD8^+^ (Hosseinzade et al., 2019).

Melittin, in combination with tumor vaccine shows immunomodulatory anti-cancer efficacy by enhancing the maturation of DC to T CD4^+^ and T CD8^+^ cells (Yang K. et al., 2023). Other insect peptides show their immunomodulatory activity by enhancing the maturation of DC and NK cells. This in turn activates T CD8^+^ cell and destroy cancer cells (Tang and Dai, 2016). Insect derived lectins show anti-cancer immunomodulatory activity through M1 macrophage mediated phagocytosis (Cao et al., 2012). The capacity of insect peptides to modify the immune response against cancer cells has been shown in several researches (Sun et al., 2014; Chen et al., 2022; Yang K. et al., 2023). It has been seen that, some insect peptide fragments interact with immune cells, including DC, natural killer (NK) cells, and T lymphocytes, among others, and enhance their activity. For instance, it has been demonstrated that certain insect peptides can increase NK cells’ cytotoxic activity, which aids in the elimination of cancer cells. Others have been discovered to support dendritic cell maturation and activation, enhancing antigen presentation and subsequent T cell activation.

## 6 Insect by-products in advanced cancer therapy and drug delivery: nanotechnology

The rise of multidrug resistance and the toxicity of standard chemotherapy drugs drives research towards targeted medicine. Efforts directed towards encapsulating and releasing anticancer medicines more effectively, raise the possibility for the nano-biomedical field to produce efficient, therapeutic, nano-sized drug delivery systems (Yao et al., 2020). Nano-oncology imparts more efficient delivery of chemotherapeutics by reducing systemic toxicity and enhancing accumulation directly to the targeted tumor microenvironment (Misra et al., 2010). Nanoparticles derived from natural compounds have been reported to have better safety profiles, revamped stability, biodegradability, and non-immunogenicity in comparison to synthetically derived nanoparticles. They also possess functional groups that can be easily modified in order to improve effcicacy. Furthermore, their high biocompatibility, hydrophilicity and lowered bio-toxicity, make bioactive components good candidates for use as nanoparticles or nano-cargo to deliver chemotherapeutics (Ion et al., 2022). A crucial aspect of nano-cargo for delivering therapeutics is their ability to target cancer cells precisely, which boosts therapeutic effectiveness while shielding healthy cells from damage (Cheng et al., 2021). Various by-products from insects have been employed for this purpose.

Silk extracted from the silkworm consists of mainly two proteins *viz.* sericin and silk fibroin (Kunz et al., 2016). The silk fibroin is an amphipathic beta sheet secondary peptide consisting of one heavy chain (365 kDa) and one light chain (26 kDa) joined together by disulfide bond. The heavy peptide chain is composed of a repetitive motif of 6 amino acids (Gly–Ala–Gly–Ala–Gly–Ser)*n* flanked by N & C terminal domains. Silk fibroin is a promising biomaterial for biomedical applications due to its distinctive structure, processing adaptability, biological compatibility, variety of biomaterial morphologies, facile sterilization, thermal resilience, surface chemistry for chemical alterations, and water solubility (Zhou et al., 2001; Jastrzebska et al., 2015; Philipp Seib, 2017), and has received formal FDA approval as a biocompatible material (Cao et al., 2017). It has been transformed to nanomaterials drug administration following its modification into films, hydrogels, coatings, capsules, micro- and nanoparticles. Fibroin nanoparticles (FNPs) can encapsulate and deliver a variety of therapeutic compounds, including small and large molecules, proteins, enzymes, vaccines, and genetic materials to target cells (Pham and Tiyaboonchai, 2020). Silk fibroin based nanoparticles are used for drug delivery by intratumoral injections and intravenous injections (Jastrzebska et al., 2015). Silk fibroin microparticles and nanoparticles have been found to be active against inflammation and its associated disorders such as arthritis and cancer (Dutta et al., 2019). Silk fibroin nanoparticles (SFNPs) loaded with chemotherapeutic drugs such as doxorubicin, cisplatin, paclitaxel, methotrexate and 5-Flurouracil proved effective at delivering the drugs and exerting anti-cancer effects *in vitro*. SFNPs loaded with paclitaxel alone or gemcitabine conjugated with SP5-52 peptide also showed anti-cancer efficacy *in vivo* in a mice model of gastric cancer and Lewis lung tumor, respectively. SFNPs loaded with binary drugs such as hydrophilic doxoribucin and hydrophobic paclitaxel were effectively internalized and inhibited the growth of cancer HeLa and HepG2 cells more effectively than when the nanoparticles were loaded with a single drug, indicating the efficiency of these nanoparticles as drug delivery systems for combination therapy. *B. mori* SFNPs loaded with curcumin were also used to deliver the phytocompound curcumin to different cell lines, resulting in significantly higher uptake and stronger anti-cancer effects in the breast cancer cell lines MCF-7 and MDA-MB-453, hepatocellular carcinoma Hep3B cells and neuroblastoma KELLY cells and HCT116 human colorectal cancer cells, compared to free curcumin. Furthermore, SFNPs loaded with plant-derived anticancer substances produced significant cytotoxic effects in cancer cells while maintaining no cytotoxicity towards healthy cells (Florczak et al., 2020).

Polyethyleneimine-modified SFNPs (PEI-SFNPs) used to co-deliver doxorubicin and survivin siRNA effectively induced apoptosis in the 4T1 mouse tumor cell line and remarkably reduced the growth rate of breast tumor in 4T1 tumor bearing mice by suppressing the survivin gene (Norouzi et al., 2021).

The silk cocoon protein, sericin, is also used in nanobiotechnology for formulating drug delivery systems. It contains hydroxyl group-containing amino acids which enable it to copolymerize with other molecules for the synthesis of novel biodegradable and biocompatible compounds that can be used for drug delivery, *in vivo* cell imaging, and other biomedical uses (Ahn et al., 2001; Cho et al., 2003; Kumar and Mandal, 2019; Elahi et al., 2021). Sericin based nanostructures can potentially deliver hydrophobic and hydrophilic chemotherapeutic drugs more rapidly into cancer cells, compared with the chemotherapeutic drug alone (Elahi et al., 2021). Sericin coated AgNO_3_ nanoparticles showed potent anticancer efficacy against the breast cancer cell lines, MCF-7 and MDA-MB-231, by inducing apoptosis, cell cycle arrest, and cancer cell specific cytotoxicity, while simultaneously reducing the side effects of the nanoparticles (Mumtaz et al., 2023; Kara et al., 2020 reported the use of nanocarrier composed of a blend of albumin and silk sericin for *in vivo* delivery of miRNA. They reported significant *in vivo* tumor integration of miR-329 and inhibition of the eukaryotic elongation factor-2 kinase (eEF2K) protein in multiple triple-negative breast cancer (TNBC) models with pronounced anti-tumor efficacy and without any side effects in mice. Albumin-sericin nanoparticles (Alb-Ser NPs) further functionalized by complexing with poly-l-lysine/siRNA and hyaluronic acid were also used for delivery of siRNA targeting casein kinase 2 (CK2), Absent, Small, or Homeotic-Like (ASH2L), and Cyclin D1 (CCND1) genes to the laryngeal cancer Hep-2 cells. The nanoparticles loaded with siRNA silenced the target genes significantly more effectively when compared with naked siRNA, resulting in significant cytotoxicity of the targeted cells (Yalcin et al., 2019).

Sericin is sensitive to pH due to its constituent amino acids with strongly polar side groups, and has been extensively studied for formulating pH-responsive delivery systems (Silva et al., 2022). In one study, folate-conjugated sericin nanoparticles were used for targeted subcellular delivery of doxorubicin to the folate-receptor-rich human oral epithelium carcinoma cell line (KB). Once inside the cells, the acidic environment of the lysosomes that contained the endocytosed nanoparticles prompted the rapid release of doxorubicin, producing significant anti-cancer effects (Huang et al., 2016). In another study, synthetic poly(γ-benzyl-L-glutamate) (PBLG) conjugated sericin micelles loaded with doxorubicin (Sericin-PBLG-DOX) induced significant cytotoxicity in adriamycin resistant MCF-7 ADR cells and HepG2 ADR cells *in vitro* as well as *in vivo* in tumor bearing nude mice transplanted with MCF-7 ADR cells and HepG2 ADR cells through enhanced cellular uptake and pH-triggered drug release (Guo W. et al., 2018). A surface charge reversal sericin-based nanocarrier used to co-deliver resveratrol and melatonin to MCF-7 breast cancer cells as combination therapy, also proved to be effective in delivering the drugs effectively and inducing apoptosis of the breast cancer cells in an acidic environment (Aghaz et al., 2023).

AgNO_3_ nanoparticles synthesized from the wings of *Mang mao* insect showed broad spectrum anti-bacterial and anti-fungal activities along with strong antioxidant efficacy (Jakinala et al., 2021). Silver nanoparticles synthesized from the defensive gland extract of Mupli beetle, *Luprops tristis* Fabricius showed potential anticancer efficacy against DLA (Dalton’s Lymphoma Ascites) cell line (Ajaykumar et al., 2023).

Mealworm insect protein was designed as a nano cargo for the delivery of curcumin to cancer cells by creating spherical biopolymer nano complexes of sizes d = 143–178 nm which are loaded with curcumin due to hydrophobic and non-covalent interaction between curcumin and insect proteins. These nano complexes enhance the release of curcumin efficiently to the cells but show moderate binding efficacy (Okagu et al., 2020). When compared to conventional approaches, the production of nanoparticles utilizing insect by-products is a more economical strategy. Natural substances found in insect byproducts such as wings, exoskeleton, and extracts serve as economical stabilizers or reducing agents during nanoparticle synthesis, lowering the total cost of production (Jakinala et al., 2021). Characteristics of some nanoparticles derived from different active components of edible insects and their modes of transfer to the cancer tissue has been listed in Table 4.

**TABLE 4:** Nanoparticles derived from bioactive components of edible insects.

Silk biomaterial	Associated drug	Particle size	Mode of transfer	Model	Results	References
*B. mori* silk fibroin film	Doxorubicin	7 mm **×** 11 mm(silk film)	Local intratumoral	Human neuroblastomaorthotopic BALB/c mice model	Combining surgical removal with a silk film device that releases doxorubicin slowly is a successful method for reducing neuroblastoma tumor development	Chiu et al. (2014)
Engineered silk fibroin-elastin miceller like nanoparticle	Doxorubicin	50, 50, 142 nm	Local intratumoral	*In vitro* HeLa cell line	The protein polymers are not cytotoxic, however the doxorubicin-loaded SE8Y nanoparticles are 1.8 times more lethal than the drug alone. Thus enhanced the anticancer effects of dox. Internalize into the cell through endocytosis	Xia et al. (2014b)
PEG/GO/SF nano composite	Doxorubicin	293.7 nm	Local	*In vitro* MCF-7 cell line	Improved cell death as compared to DOX alone. PEG/GO/SF/DOX releases more drug in the acidic environment that simulates tumor tissue. It also has good drug entrapment and loading efficiency	Jeshvaghani et al. (2023)
Silk fibroin NP surface modified with cRGD	naphthalene diimide derivative	<100 nm	Local	Glioma cell line U373and D384	Deliver the drug to the target active site-specific tumor cells	Pirota et al. (2023)
GO-CMC hydrogel/SF/Fe3O4 Nanobiocomposite	-	Hydrogel	Local	*In vitro* BT549 cancer cell line	The nanobiocomposite did not affect the normal HEK293T cells while induce death to BT549 cells and act as potent anticancer agent	Ghafori Gorab et al. (2022)
Silk fibroin NP-CM prepared by solution-enhanced dispersion by supercritical CO2	Curcumin	<100 nm	Local	*In vitro* HCT116 colon cancer cells	Effects on normal epithelial cells reduced and the anticancer efficacy of curcumin increased	Xie et al. (2016)
Silk fibroin –polyvinyl alcohol	Doxorubicin	600–1,800 nm	Intravenous via tail vein	BALB/c nude mice xenogratted with MDA-MB-231	Excellent monodispersity high effectiveness of the drug encapsulation; 72 h controlled medication release. Drug release was induced and accelerated by external ultrasound	Cao et al. (2017)
SF-PVA	Doxorubicin	2.8–6.8 µm		KELLY Neuroblastoma cells	High efficiency and capacity for loading drugs medication release that is pH-dependent. Medication release that continues for 23 days.THP-1 monocyte uptake Macrophage activation upon exposure to silk particles	Florczak et al. (2020)

## 7 Limitations of natural products in clinical trials

Crucial aspects of the drug development process include identification and characterization of the active ingredients in natural products, and guaranteeing their stability and consistency. Current methods for drug discovery in contemporary medicine prefer single compound-based treatment over crude extracts of natural products. However, given the multifaceted nature of many ailments including cancer, it is not surprising that the search for effective treatments has not been successful when relying solely on single compounds (Thomford et al., 2018).

While natural products contain several bioactive components, the complexity of the molecular combinations from natural sources makes the search for novel therapeutic possibilities challenging, There are also several limitations to the use of natural products derived from insects and other sources in clinical trials. These include: (i) *In vitro* preclinical research frequently entails prolonged exposure to elevated quantities of a target natural substance. In humans, this kind of exposure is usually not achievable, especially when it comes to oral drugs that may have a restricted bioavailability; (ii) Safety and allergic reactions is a crucial subject for translation of nature-derived products to human subjects; (iii) insects might acquire diseases or contain residues of pesticides and heavy metals from their natural ecosystem which may raise safety issues; (iv) preclinical efficacy does not always translate into human success, and no dietary supplement research has yet received regulatory clearance; (v) the lack of established protocols for evaluating natural products in preclinical and clinical research is another significant barrier (Paller et al., 2016; Thomford et al., 2018).

Changes in the experimental setting, such as assay methods, dosing regimens, and primary extraction processes, can lead to contradictory findings and reduce the comparability of data from many trials. Establishing guidelines for the description, preparation, and evaluation of natural products can improve the reliability of study results and facilitate regulatory approval of clinical trials (Andrade et al., 2016).

## 8 Future perspectives

Insects have been traditionally consumed as food and used for the preparation of folk medicine by several populations, globally. Due to rapid growth in population and rising food demand, it is imperative that sustainable, nutrient-dense, and environmentally sustainable alternative food sources explored. Entomophagy has the potential to solve difficulties with nutrition, sustainability, and global food security because insects possess high nutritional value and insect farming is economically sustainable, generates a significant amount of edible biomass with far lower land, water, and feed requirements, and also has a low emission of greenhouse gases (Shah et al., 2022).

Insect extract and insect venom are crucial and time-tested components of complementary and alternative medicine, having been applied and improved by physicians over several generations for treating various disorders (Wainwright et al., 2022). There is strong evidence to support the rising use of insect-derived products and crude extracts as a useful component of treatment and a profusion of experimental evidence demonstrating their vast diversity being used to successfully cure various types of ailments and malignancies. Due to their antioxidant and anti-inflammatory activities, insect extracts and their byproducts have the ability to improve cancer treatment in addition to successfully reducing side effects and symptoms (Dutta et al., 2019). Indeed, various studies indicate that insect-derived bioactive components such as cecropin, sericin and mellitin exhibited significant cytotoxicity in cancer cells, while producing no cytotoxicity in normal cells. This selective action could significantly reduce the unwanted side effects associated with chemotherapy, and warrant further investigations into the cellular mechanisms involved. Traditional medical systems from many cultures have long acknowledged the therapeutic benefits of edible insects and the substances they produce. Through the integration of this age-old knowledge with contemporary scientific discoveries, we can open up new avenues for the development of trustworthy anticancer treatments. Both conventional wisdom and bioactive substances obtained from insects have great promise as trustworthy anticancer treatments. By funding research and fusing traditional knowledge with contemporary technology, we can create efficient medications that are accessible, inexpensive, and sustainable on a global scale. To overcome the limitations related to the use of natural products in clinical trials, more focus is required on establishing standardized protocols for the extraction of bioactive compounds and assessment of the efficacy of natural products. It is also crucial to have strict preclinical and clinical evaluation methodologies in place. As these bioactive compounds show higher synergistic anticancer efficacy in conjugation with other nature derived bioactive compounds or in combination with chemotherapeutic agents, more research should focus on combination therapy. For example, cecropin shows higher anticancer effects in combination with curcumin (Mahmoud et al., 2022); and, a hybrid vaccine developed by conjugating mellitin with RADA32 (cell assembling peptide), CpG (immune adjuvant) and tumor lysate shows strong antitumor immunological activity in melanoma B16-F10 xenograft mice (Yang K. et al., 2023). There should also be more focus on the mode of delivery of chemotherapeutic agents and/or natural products to the target tumors or cancer sites in order to reduce systemic toxicity and improve therapeutic efficacy. In this context, nano-cargo delivery systems based on insect-derived bioactive components such as silk fibroin and sericin protein hold great promise and should be extensively explored.

## 9 Conclusion

This evidence based review demonstrates the anti-cancer and immunomodulatory efficacy of edible insect derived extracts and/or bioactive compounds. Edible insects are rich in nutrients such as proteins, amino acids, minerals, vitamins and fatty acids. Additionally, edible insects contain a wide range of useful compounds, including bee venom components; silk cocoon and antimicrobial peptides which possess anticancer immunomodulatory activity. Bioactive components derived from insects act as potent nano-cargo to deliver the therapeutics directly to the tumor microenvironment by lowering the systemic side effects of chemotherapeutic drugs.

Focused research would hasten the development of novel, potent insect-derived therapeutics and drug delivery systems against cancer. However, detailed investigations into the toxicological effects, if any, of these bioactive components, as well as their capacity to traverse the blood-brain barrier, their bioavailability, skin permeability, lipophilicity, and pharmacodynamic qualities, are required. It is also imperative to establish guidelines and standardized protocols for the extraction and preparation of natural products derived from insects, as well as for the evaluation of their efficacy in preclinical and clinical studies. These strategies will enable the transition of significant preclinical findings to translational research, so that the therapeutic potential of insect-derived bioactive components may be fully explored.
